# Supplementary material for: Identification of photoperiod-induced specific miRNAs in the adrenal glands of Sunite sheep (Ovis aries)
Source: Front Vet Sci. 2022 Jul 22;9:888207. doi: 10.3389/fvets.2022.888207 (PMC9354845; doi:10.3389/fvets.2022.888207)
Supplement: Supplementary file 1 [file Data_Sheet_1.ZIP › Supplementary file/miRDeep2 file.zip!/miRDeep2 file/Novel_Structure/LP42A1/Novel_235.pdf]

Provisional ID	:	NC_019469.2_20277
Score total	:	0.1
Score for star read(s)	:	-1.3
Score for read counts	:	0
Score for mfe	:	-0.1
Score for randfold	:	1.6
Score for cons. seed	:	
Total read count	:	103
Mature read count	:	103
Loop read count	:	0
Star read count	:	0

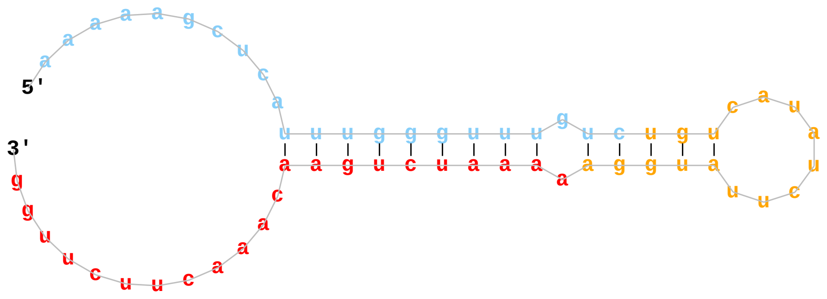

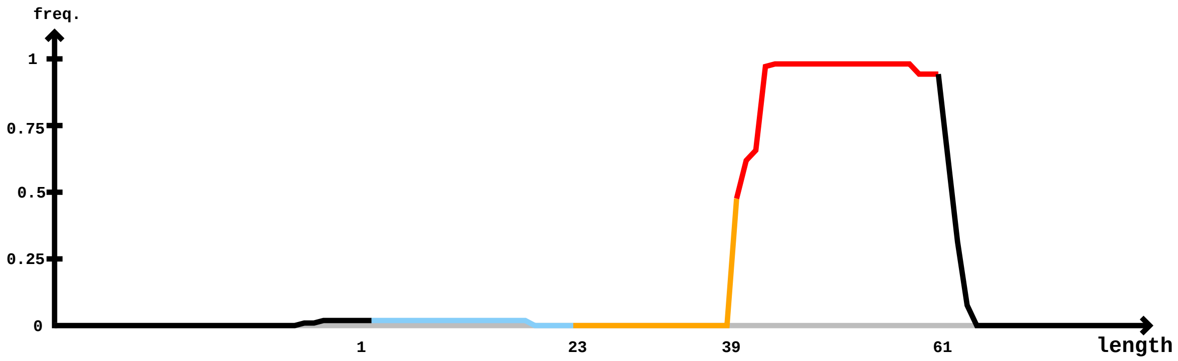

Star Mature

```
5'- cuggacaaaaaaagauuauauugaguugguc aaaaagcucauuuggguuuguc ugucauaucuuaugga aaaaucugaacaaacuucuugg ccaauccagugugaaacacag   -3'    exp
    ...............((((((((.(((((((((.(((....(((((((((.(((((........))))).)))))))))....))).))))))))).)))))))).......    reads    mm    sample
    ........................guuggCcaaaaagcucauuugggu................................................................    1        1     seq
    ..........................uggCcaaaaagcucauuugggu................................................................    1        1     seq
    .....................................................................aaaaucugaacaaacuuUu........................    4        1     seq
    .....................................................................aaaaucugaacaaacuuUuugg.....................    45       1     seq
    .....................................................................aaaaucugaacaaacuuUuuggc....................    1        1     seq
    ......................................................................aaaucugaacaaacuuUuugg.....................    14       1     seq
    ......................................................................aaaucugaacaaacuucuuggc....................    1        0     seq
    .......................................................................aaucugaacaaacuuUuuggc....................    4        1     seq
    ........................................................................aucugaacaaacuuUuuggcc...................    25       1     seq
    ........................................................................aucugaacaaacuuUuuggcca..................    8        1     seq
    .........................................................................ucugaacaaacuuUuuggc....................    1        1     seq
```
